# Pediatric Canadian Triage and Acuity Scale (PaedsCTAS) as a Measure of Injury Severity

**DOI:** 10.3390/ijerph13070659

**Published:** 2016-07-07

**Authors:** Morgan Thorn Yates, Takuro Ishikawa, Amy Schneeberg, Mariana Brussoni

**Affiliations:** 1 School of Population and Public Health, University of British Colombia, Vancouver, BC V6T 1Z4, Canada; morgan_yates13@hotmail.com (M.T.Y.); amyschneeberg@gmail.com (A.S.); 2 Department of Pediatrics, University of British Colombia, Vancouver, BC V6T 1Z4, Canada; tishikawa@cfri.ca; 3 British Columbia Injury Research & Prevention Unit, British Columbia Children’s Hospital, F508-4480 Oak Street, Vancouver, BC V6H 3V4, Canada

**Keywords:** hospitalization, length of stay, accident, surveillance, Health Related Quality of Life

## Abstract

This research explored whether the pediatric version of the Canadian Triage Acuity Scale (PaedsCTAS) represented a valid alternative indicator for surveillance of injury severity. Every patient presenting in a Canadian emergency department is assigned a CTAS or PaedsCTAS score in order to prioritize access to care and to predict the nature and scope of care that is likely to be required. The five-level PaedsCTAS score ranges from I (resuscitation) to V (non-urgent). A total of 256 children, 0 to 17-years-old, who attended a pediatric hospital for an injury were followed longitudinally. Of these children, 32.4% (*n* = 83) were hospitalized and 67.6% (*n* = 173) were treated in the emergency department and released. They completed the PedsQL^TM^, a validated measure of health related quality of life, at baseline (pre-injury status), one-month, four- to six-months, and 12-months post-injury. In this secondary data analysis, PaedsCTAS was found to be significantly associated with hospitalization and length of stay, sensitive to the differences between PaedsCTAS II and III, and related to physical but not psychosocial HRQoL. The findings suggest that PaedsCTAS may be a useful proxy measure of injury severity to supplement or replace hospitalization status and/or length of stay, currently proxy measures.

## 1. Introduction

As the leading cause of mortality and one of the leading causes of morbidity in the United States and Canada, childhood injury is a significant public health issue [1,2]. An estimated 9.2 million children in the U.S. and 681,000 children in Canada visit an emergency department (ED) for an unintentional injury annually [1,3]. Therefore, measures of injury severity are useful to health professionals and researchers in order to track and better understand injuries and work towards preventing them. Current indicators of injury severity range from general measures, such as hospital admission and length of stay, to injury specific measures such as the Injury Severity Score [4]. General measures are readily available through hospital databases, but are not injury specific, and there are questions about how well they predict injury severity [4]. Injury specific measure are more accurate, but they are complex and time consuming to calculate so are not routinely calculated [4]. An easily accessible and relatively accurate measure of injury severity, that does not require additional calculation, would therefore be of benefit.

Implicit in the use of hospital admission and length of stay as measures of injury severity is the assumption that injury severity is the primary factor predicting hospitalization status and that length of stay is standardized for similarly severe injuries. However, several extraneous factors influence both variables. The decision to admit, and average length of stay for similar injuries, can change over time with modifications to clinical practice [4,5]. Lower hospitalization rates have been observed in urban compared to rural areas, as outpatient options are more readily accessible [4,6]. Other factors, such as the availability of hospital beds, parents’ concerns and ability to manage care at home, the child’s coping, and hospital acquired infections, can also influence admission and the length of stay [5,7].

Health Related Quality of Life (HRQoL) measures are rarely used as indicators of injury severity because they are not routinely collected; yet they can provide key data on children’s post-injury recuperation and outcomes [8,9,10]. Children’s HRQoL scores generally decrease in the first few months post injury, then return to baseline [10,11,12]. Hospitalization and length of stay have been associated with HRQoL post-injury in pediatric populations [10]. Measures of HRQoL generally include physical and psychosocial aspects, however, when looking at HRQoL and injury severity, the physical aspects have greater bearing on determining the physical severity of the injury than psychosocial aspects. The psychosocial aspects of HRQoL can vary greatly between children with the same condition [13] and seem to have little relationship with injury or illness severity in a variety of conditions [13,14]. Tessier et al. (2014) speculated that this may be due to social interaction, and school environments playing more of a role in determining psychosocial HRQoL [13]. Regardless of why HRQoL is unrelated to injury severity, a general measure of injury severity should primarily relate to physical, rather than psychosocial, aspects of HRQoL.

The pediatric version of the Canadian Triage Acuity Scale (CTAS), called the PaedsCTAS may represent an alternate indicator for surveillance of injury severity. Every individual presenting in a Canadian ED is assigned a CTAS or PaedsCTAS score in order to prioritize access to care and to predict the nature and scope of care that is likely to be required [15]. The five-level PaedsCTAS score ranges from I (resuscitation) to V (non-urgent) [16]. PaedsCTAS level I is assigned to any individual with a condition that represents an immediate threat to life or limb, while PaedsCTAS II conditions are also potentially life threatening but pose a less imminent threat. PaedsCTAS III children present with symptoms that are potentially serious, but are not likely to be life threatening. PaedsCTAS IV and V are generally less severe conditions with no threat to life or limb. The PaedsCTAS is an ordinal scale in that differences between adjacent scale values do not represent equal intervals. The PaedsCTAS score is assigned by highly trained ED nurses [15] based on the initial impression of illness severity, an evaluation of the presenting complaint, and an assessment of behavior and physiological measurements [17]. The nurses assigning the score have extensive experience working in the ED, are trained in the use of the PedsCTAS assessment, and typically complete a refresher course every few years [16].

PaedsCTAS has been associated with hospitalization, health resources use and length of stay [16,18,19]. Therefore it is hypothesized that PaedsCTAS will also be associated with injury severity, though the use of a triage scoring system as a measure of injury severity has not been extensively investigated [20,21,22]. The PaedsCTAS may provide useful information for surveillance of injury severity as it is a valid tool for triage assessment of children [18,23], is used for all presentations at an ED, not just for injuries, and is mandated as an essential piece of information to be collected in all Canadian EDs, so it is standardized and commonly available [15]. Furthermore, extraneous factors that have been associated with hospitalization and length of stay, mean that PaedsCTAS may be a more valid proxy measure for routine injury surveillance then these two measure, which are currently commonly used as proxy measures of injury severity. Therefore, the objective of this study is to assess whether PaedsCTAS is a useful and valid proxy measure of injury severity by examining the relationship between PaedsCTAS and hospital admission, and length of stay, currently used proxy measures. Also by examining the relationship between PeadsCTAS and HRQoL at one, four and 12 months post injury, as this measure adds a more contextual understanding of injury severity.

The primary outcome of interest was whether PaedsCTAS was associated with hospitalization, length of stay, and HRQoL, in order to determine if PaedsCTAS was a valid proxy indicator of injury severity. This was of interest as there are a number of concerns with hospitalization and length of stay as proxy measures of injury severity. Therefore, the analyses explored suitability of the PeadsCTAS to potentially replace hospitalization and length of stay in the surveillance of injury severity.

## 2. Methods and Materials

### 2.1. Sample 

Children between the ages of 0 and 17 presenting to British Columbia Children’s Hospital (Canada) between February 2011 and December 2013, with an injury as their principal diagnosis were eligible for inclusion. British Columbia Children’s Hospital emergency department is the only Level 1 pediatric trauma center in the province of British Columbia, Canada, and sees over 43,000 children every year. A convenience sample of injured children and their parents, or primary caregivers, were approached in the ED or on the hospital wards by a research assistant. Eligible children had an injury as their primary diagnosis, resided in British Columbia, had caregivers that were English-speaking, and had caregivers who agreed to complete a number of surveys. Study recruitment deliberately over-sampled hospitalized children. Parents provided written consent, and children aged 7 to 16 years provided assent to participate. Parents of all children completed a baseline survey, as did children aged 8 or older. A $2 gift card honorarium was provided to each participant for survey completion at each time point.

Baseline data included questions regarding the injury and sociodemographic information including age, gender, language spoken at home, family’s gross income, parents’ education levels and work status. The child’s state of health prior to the injury was assessed using the Pediatric Quality of Life Inventory (PedsQL^TM^) [24]. PedsQL^TM^ is a measure of HRQoL which has been used in many pediatric health studies which has been shown to demonstrate validity, reliability, sensitivity and responsiveness for child self-reported quality of life [24]. Follow-up surveys, which included the PedsQL^TM^, were sent out by mail and email at one-month, four- to six-months and 12-months post-injury. Additional data were abstracted from the child’s hospital chart, including PaedsCTAS score, whether the child was hospitalized and length of stay. The PaedsCTAS score was assigned by the original triage nurse in the emergency department, not as part of the study. The Statistics Canada’s Postal Code Conversion File Plus (PCCF+) was used to link the children’s home address with a proxy socioeconomic status (SES) measure for their neighborhood, the Quintile of Adjusted Income per Person Equivalent (QAIPPE). This is a measure of after-tax income per person but adjusted for household size, which is divided into fifths to create income quintiles [25]. This measure was previously used in health research in Canada [25]. The study protocol was approved by the Children’s and Women’s Health Centre of British Columbia Research Ethics Board (H09-01627). Details regarding the study methods are available elsewhere [26].

### 2.2. Statistical Analysis

This study was a secondary analysis of this dataset, and all statistical analyses were done using IBM SPSS Version 22 [27]. As PaedsCTAS is not currently used as an indicators of injury severity, extensive effort was made to determine a priori which analysis would best demonstrate this relationship. The analyses conducted herein were therefore meant to examine an overall relationship between PaedsCTAS and hospitalization and length of stay. Distributions and frequencies were calculated for all continuous and categorical variables, respectively. Analyses examined first whether the children’s PaedsCTAS scores are sensitive to the differences between PaedsCTAS II and III. Logistic regression was used to examine this, with hospitalization status as the independent variable and PaedsCTAS II and III as the dependent variable. This analysis was done to show whether the PaedsCTAS tool was indeed sensitive to very slight differences in severity as clinically PaedsCTAS II and III have the least difference in severity between levels in the PaedsCTAS scale.

It was decided a priori that sample sizes in the PaedsCTAS I and IV categories were likely to be small. Therefore, the majority of the analyses would be conducted with the sample collapsed into three groups, separated based on clinical similarity (PaedsCTAS I and II, PaedsCTAS III, and PaedsCTAS IV and V). Analyses explored (a) the relationship between PaedsCTAS and hospitalization status and length of stay; (b) the relationship between PaedsCTAS and demographic variables, including age and SES; and (c) comparison of PaedsCTAS, hospitalization and length of stay as predictors of change in HRQoL one year post-injury. Logistic regression and chi-squared analysis were used to explore the relationship between PaedsCTAS level and hospitalization status. Then logistic regression analysis was used to examine the relationship between PaedsCTAS and length of stay. To examine the demographic variables, Chi squared analysis was conducted to determine if PaedsCTAS was associated with child gender and SES, as these variables are known to be associated with hospitalization and length of stay. All analysis methods and variables to include in the analysis were determined a priori, based on specific research questions. Therefore, univariate analysis was used to understand each variable individually, rather than to determine which variables to include in the models. Multivariable models including all the demographic variables and hospitalization or length of stay were also run to further understand the relationship of the PaedsCTAS to hospitalization and length of stay.

PaedCTAS, hospitalization status, and length of stay have different measurement levels, for instance continuous numeric or categorical. Therefore it was not possible to directly assess how much these measure agree [28], hence, we proposed an indirect approach. Longitudinal analysis with generalized estimating equation (GEE) models was conducted to compare how well hospitalization, length of stay, and PaedCTAS predicted HRQoL during the year following the injury. Data were taken from surveys completed by both children, depending on their age, and their parents, at one month, four months and 12 months post injury. Children were included in this analysis if baseline data, plus survey data at one other time point (one, four or 12 months) were available. Therefore, this analysis has a slightly smaller sample size of 185 children because not all children included in the other analysis had follow up data available. All available survey data were included, if the child has survey data at multiple time points. To account for time-dependent changes in PedsQL^TM^ scores, all models included time from baseline as covariate and were fitted in the same data set. Three sets of three longitudinal models each, were each fitted in the same data set (nine models in total); set 1: Three models, each predicting the physical, psychological, and total PedsQL^TM^ scores, using PaedCTAS and time as covariate and factors, respectively; set 2: Three models, each predicting the physical, psychological, and total PedsQL^TM^ scores, using hospitalization and time as covariate and factor, respectively; set 3: Three models, each predicting the physical, psychological, and total PedsQL^TM^ scores, using length of stay and time as covariates. Each set of models were identical to one another, except in the proxy measure of injury severity (*i.e.*, PaedCTAS, hospitalization status, length of stay). We reasoned that if the model with PaedCTAS has a similar or better fit compared with each of the other models, then PaedCTAS could be considered at least as good a proxy measure of injury severity as hospitalization and length of stay.

## 3. Results

A total of 928 children were initially approached for this study, with 378 of these children enrolling and completing a baseline survey. From this sample, children who had a preexisting health condition, whose injury was the result of deliberate violence, or who were found not to have an injury after treatment were excluded, leaving 351 children. During data cleaning some children’s data were found to be missing baseline PedsQL score, residential postal codes, or PaedsCTAS scores and were therefore excluded. Of these 81 children were excluded due to missing PaedsCTAS scores. Changes were made to how hospital electronic records were kept during the beginning of study, so PaedsCTAS score were only available after this change. The baseline sample included 256 children and was used for initial analysis as well as linear regression, logistic regression and chi squared analysis. The GEE analysis included 185 children who completed both the baseline survey as well as one follow-up survey (at 1 month, 4–6 months or 12 months post injury). For reference, 135 children completed all four surveys (baseline, 1 month, 4–6 months and 12 months). The sample of children who agreed to participate in the study but who were excluded later due to incomplete data were not statistically different from the baseline sample on any of the available demographic or injury variables in the dataset. This sample is slightly different than the sample used for other articles using this dataset, due to different inclusion criteria [26].

The total study sample consisted of 256 children who ranged in age from 0 to 17 when their injury occurred (mean age 8.2 years). Of these children, 32.4% (*n* = 83) were hospitalized and 67.6% (*n* = 173) were treated in the ED and released. The most common PaedsCTAS score was IV (49.2%, *n* = 126). When PaedsCTAS score was examined by hospitalization status and length of stay, it was found that the lower the PaedsCTAS scores, the higher the proportion of children being admitted. All of the PaedsCTAS I were admitted whereas only two (1.6%) of the PaedsCTAS IV children were admitted. Of the nine children classified in the PaedsCTAS V category, three (33.3%) were admitted. This was likely due to the nature of BCCH as a level 1 trauma center, as 38 children (14.8%) were transferred directly to BCCH from other hospitals and 15 (5.9%) had been directed to come to BCCH ED to be seen by a specialist.

The logistic regression reported in Table 1 was conducted in order to determine if likelihood of hospitalization varied between PaedsCTAS scores of II and III. Children rated as PaedsCTAS II had greater odds of hospitalizations relative to children who had a PaedsCTAS score of III.

Table 2 gives the counts associated with the demographic variables and provides Chi Squared and Fisher’s exact test for each of these variables. Gender and socioeconomic status were not statistically significantly associated with PaedsCTAS. Age did not appear to be significantly associated, though the *p* value was too close to make a conclusive determination.

Table 2 also shows that hospitalization was associated with PaedsCTAS and that children rated as PaedsCTAS I and II, and those rated as PaedsCTAS III, were more likely to be hospitalized than those rated PaedsCTAS IV and V. PaedsCTAS was also associated with the length of stay in hospital. The number of days in hospital decreased as the PaedsCTAS score increased.

Table 3 shows that even with the covariates included in the model, the only variables which are associated with PaedsCTAS are hospitalization and length of stay.

Table 4 presents goodness of fit for all nine models (three sets of three models), where PaedsCTAS, hospitalization status, and length of stay were compared in terms of how well they predict PedsQL Total, PedsQL Physical, and PedsQL Psychosocial scores during the year following the injury. The Corrected Quasi Likelihood under Independence Model Criterion (QICC) indicates model fit, with a lower number indicating a better fit. Results indicate that hospitalization status and length of stay are associated with PedsQL Total, PedsQL Physical, and PedsQL Psychosocial scores. However, PaedsCTAS was significantly associated only with PedsQL Physical, but not with PedsQL Total and PedsQL Psychosocial. For reference, the 185 children in this analysis completed a baseline survey and a survey at one other time period. A total of 135 children completed all four surveys (baseline, 1 month, 4–6 months and 12 months).

## 4. Discussion

Hospital admission and length of stay are accepted proxy measures for injury severity [4,7], but are influenced by extraneous clinical and non-clinical factors, such as child gender and socioeconomic status [29,30,31]. In contrast, PaedsCTAS was not statistically significantly associated with either child gender or socioeconomic status, either in univariate or multivariate models. The analysis in this study showed a marginally non-significant association between PaedsCTAS and child age. Previous research has found associations noted between child age and hospital admission and length of stay [31]. 

Hospital admission and length of stay were associated with physical and psychosocial HRQoL. Hospitalization can be highly stressful for children, due to the injury they sustained, changes to their daily routine and separation from parents and siblings [32]. These stressors may explain the association between psychosocial HRQoL and hospitalization and length of stay that was evident in our sample. However, psychosocial aspects of HRQoL have been found to vary greatly between children with the same condition [13], and to have little relationship with injury or illness severity in a variety of conditions [13,14,33]. Therefore, as hospitalization and length of stay are themselves related to psychosocial quality of life, using them as measures of injury severity to understand, for example, how severity of injury affects long-term functioning of children, greatly increases the risk of a type 1 error. Additionally, previous research has shown that psychosocial HRQoL post injury is not related to injury severity generally [34]. While this may seem counter intuitive, many other factors, such as long term impact of injury and visible scarring are speculated to have a greater effect on HRQoL then injury severity [13]. Additionally, Tessier et al. (2014) speculated that social interaction, and school environments play more of a role in determining psychosocial HRQoL [13].

Because the PaedsCTAS is only related to physical aspects of HRQoL and is not related to demographic variables, it may be a more appropriate indicator of injury severity, as it is not influenced by other factors that are known to be unrelated to injury severity. PaedsCTAS was also significantly associated with the likelihood of hospital admission, and sensitive to the difference in PaedsCTAS scores II and III. Taken together, the evidence suggests that PaedsCTAS could be a more appropriate proxy measure of injury severity in children than hospitalization or length of stay, though this study does not provide a direct correlation between injury severity and PaedsCTAS score.

No injury severity indicator is perfect, Kim (2012) highlighted that many of the current measures and proxy measures are time consuming, require specialized knowledge, or in-depth patient assessments to calculate [35]. Beattie et al. (1998) also noted that current measures provided limited ways of objectively scoring non-trauma related injuries, like poisoning, choking, inhalation or near drowning [4]. PaedsCTAS is used for all presentations at an ED, not just for injuries. Therefore it captures all injuries including events such as poisoning and near drowning, which other injury severity measures do not capture [4]. Kim (2012) adds that any injury severity indictor used should be accurate, reliable and specialized in measuring what it purports to measure [35]. PaedsCTAS has been found to be easily replicated and has good interrater reliability [36,37,38], making it accurate and reliable. Extensive research has established its validity for triage assessment of children [18,23]. An added advantage of using PaedsCTAS as a proxy measure of injury severity is that it is highly sensitive. Analysis was conducted that showed PaedsCTAS is sensitive enough to detect a difference in hospitalization rates between children given a PaedsCTAS score of II and of III. All children given a PaedsCTAS score of I were hospitalized, whereas only 82% of those given a score of II and 38.2% of those given a score of III were hospitalized. Therefore the ability of the PaedsCTAS score to show a higher odds of hospitalization in the PaedsCTAS score of II vs. III, showed the sensitivity of this measure. Further, it is mandated as an essential piece of information to be collected in all Canadian EDs [15], making it readily available. The fact that it is collected for all ED presentations also allows for easy comparisons to be made between injuries and other conditions. Unfortunately, ParedsCTAS does require specialized training to calculate and is somewhat time consuming, however as it is always calculated in the ED, it is unlikely that it would be a need to be recalculated at a later date. Overall, PaedsCTAS meets the majority of the minimal requirements identified for any measure or proxy measure routinely used to calculate injury severity, as well as being sensitive to the difference between various PaedsCTAS scores and significantly associated with hospitalization and length of stay; making PaedsCTAS an appropriate proxy measure of injury severity.

### Limitations

Analyses may have been influenced by an over-representation of hospitalized children and of some PaedsCTAS scores in this dataset, and the non-normal distribution of the length of stay variable. Hospitalized children were deliberately over sampled to be used for other analysis not included in this article. In a large study of 12 Canadian Pediatric EDs by Gravel et al. (2013) [19], involving 550,940 children, PaedsCTAS I was 0.6% of the sample, PaedsCTAS II 11%, PaedsCTAS III 37%, PaedsCTAS IV 7% and PaedsCTAS V 0.4%. So the sample for this study was over-representative of PaedsCTAS I, IV and V, while under representing PaedsCTAS III. Further, the study by Gravel et al. (2013) [19] had an admission rate of 15%, while this study sample had an admission rate of 32%, more than double. Analyses were also influenced by the fact that length of stay is not normally distributed. This is likely the case for all studies that look at length of stay (such as Reference [19]), and not unusual in this sample. While it is possible that the sampling distribution limited the current analysis, it was also beneficial as it created a dataset with a wide variety of injury types and severities, which allowed for additional analysis. Lower PaedsCTAS score are much more common in random samples [19], however, having a more diverse sample also gave more insight into injury severity and PaedsCTAS scores.

The relationship between child age and PaedsCTAS score is complex as higher PaedsCTAS scores may be assigned for some injuries in children under one year, as children’s conditions in this age group can change rapidly. This may be an explanation for why the *p*-value of the chi squared analysis of the relationship between children age and PaedsCTAS score was close to the significance level, causing somewhat inconclusive results. There were only seven children younger the 12 months included in this study. It is possible that a larger sample in this age category would have influenced the results. 

Approximately 15% of the sample did not initially present at BCCH for their injury. Therefore, it is likely that the PaedsCTAS score of these children recorded in the BCCH records did not acutely reflect their initial injury presentation as these children had already been assessed by a physician and stabilized at another hospital. The PaedsCTAS guidelines acknowledge that patients previously assessed for the same concern, transferred for admission, or for specialist care, can be hard to assign a PaedsCTAS level [17]. There was also no way for the authors to determine what the initial PaedsCTAS score was from the original presentation at another hospital. Overall this does somewhat limit the PaedsCTAS data, and is noted as a caution when interpreting the data.

Hospitalization and length of stay are not direct measures of injury severity, so it is hard to conclusively say that PaedsCTAS is generally a strong measure of injury severity based on this study. We recommend future studies compare PaedsCTAS with more direct measures of injury severity, such as the Injury Severity Score and the Abbreviated Injury Severity Score. Additionally, as this type of study has never been conducted before, the statistical analysis was chosen on based on the authors’ reasoning and the data available. The use of QICC to compare analysis different exposure variables may be a limitation, but serves as a preliminary analysis to be confirmed in future research. We strongly encourage further investigations into this question.

## 5. Conclusions

Hospitalization and length of stay are routinely used as proxy measures of injury, but there are a number of issues with using these measures. Hence the need for a better way of routinely tracking injury severity, which PaedsCTAS offers. PaedsCTAS scores were not statistically significantly influenced by gender or socioeconomic status, but were statistically significantly associated with hospital admission, were highly sensitive to difference in PaedsCTAS scores and were only related to the physical aspects of quality of life, not the psychosocial aspects. PaedsCTAS has been shown to be a valid tool for triage assessment of children [18,23], it is used for all presentations at an ED, not just for injuries, and its collection is mandated in all Canadian EDs [15]. PaedsCTAS is however not an injury specific measure, indicating that its primary purpose will be as a proxy measure of as an injury severity measure for surveillance. Though this study does not provide a direct correlation between injury severity and PaedsCTAS score, overall, PaedsCTAS is a more appropriate, more practical, proxy measure of injury severity than hospitalization or length of stay.

### What is already known on this subject

- Hospitalization and length of stay are often used as proxy measures of injury severity because they are routinely available in administrative datasets.
- These measures are influenced by non-clinical factors such as gender and socioeconomic status.
- PaedsCTAS is a highly standardized triage score assigned to all patients attending Canadian hospitals.

### What this study adds

- PaedsCTAS is not statistically significantly associated with gender or socioeconomic status, but is statistically associated with hospital admission and length of stay.
- PeadsCTAS is associated with physical, but not psychosocial health-related quality of life.
- PaedsCTAS may be a more appropriate proxy measure of injury severity than hospitalization and length of stay and is routinely collected in administrative datasets.

## Figures and Tables

**Table 1:** Logistic regression modeling of hospitalization by PaedsCTAS.

		Variable	*p*	Odds Ratio or Slope	95% Confidence Interval
Hospitalization (logistic regression)	Paeds CTAS II and III Scores (*n* = 105)	PaedsCTAS II	0.000	7.38	(3.00, 18.21)

**Table 2:** Demographics by PaedsCTAS score with Chi squared or Fisher’s exact analysis.

Variables	PaedsCTAS Score		
	I and II	III	IV and V
Sex N (percent of sample)	66 (25.8)	55 (21.5)	135 (52.7)
Boys	46	31	83
Girls	20	24	52
Chi Squared *p* Value	0.3009		
Age Category			
Less than 13 months	1	5	1
13 months to 2 years	2	2	10
2 to 5 years	13	11	32
5 to 8 years	20	12	22
8 to 13 years	17	16	44
Greater than 13 years	13	9	26
Chi Squared *p* Value	0.0560		
Quintile of Annual Income Per Person Equivalent (QAIPPE)			
QAIPPE 1—lowest income	8	5	24
QAIPPE 2	10	6	14
QAIPPE 3	12	11	29
QAIPPE 4	16	10	27
QAIPPE 5—highest income	20	23	41
Chi Squared *p* Value	0.6638		
Hospitalization Status			
Hospitalized	57	21	5
Not Hospitalized	9	34	130
Chi Squared *p* Value	0.000		
Length of Stay (LOS)			
Not Hospitalized	9	34	130
Less than 1 day	5	4	2
1–3 days	26	10	2
4–7 days	9	3	1
8–14 days	12	2	0
More than 14 days	5	2	0
Fisher’s Exact Test *p* Value	0.000 *		
Injury Category			
Major trauma	2	0	0
Head trauma	2	2	0
Spinal fracture	4	11	8
Internal organ injury	3	2	1
Burn major	0	2	1
Hand or foot amputation	6	0	0
Head injury	10	14	12
Ingestion/choking	21	1	0
Lower extremity fracture	3	13	79
Upper extremity fracture	3	0	0
Minor external injury	12	10	34
Fisher’s Exact Test *p* Value	0.000 *		
Transferred to BCCH from other facility	13	14	11
Referred for follow-up visit with specialist	1	3	11
Repeat visit for a previous injury	1	2	16
First visit for this injury	51	36	97
Fisher’s Exact Test *p* Value	0.001 *		

* = significant at 0.05.

**Table 3:** Logistic regression model with all covariates.

Variable	*p*	Odds Ratio	95% Confidence Interval
Hospitalization (yes)	0.000 *	0.017	(0.008, 0.036)
Sex (male)	0.240	0.696	(0.378, 1.270)
QAIPPE 1	Reference	Reference	Reference
QAIPPE 2	0.107	0.385	(0.118, 1.213)
QAIPPE 3	0.612	0.768	(0.271, 2.109)
QAIPPE 4	0.664	0.792	(0.271, 2.247)
QAIPPE 5	0.444	0.688	(0.257, 1.756)
Age Category			
<1 year	Reference	Reference	Reference
13 months to 2 years	0.551	0.569	(0.087, 3.700)
2 to 5 years	0.120	3.706	(0.683, 19.885)
5 to 8 years	0.336	2.244	(0.416, 11.944)
8 to 13 years	0.748	1.305	(0.246, 6.771)
>13 years	0.183	3.144	(0.564, 17.439)
LOS Less than 1 day	0.000 *	0.040	(0.010, 0.141)
1–3 days	0.000 *	0.0178	(0.007, 0.043)
4–7 days	0.000 *	0.020	(0.005, 0.074)
8–14 days	0.000 *	0.006	(0.001, 0.026)
More than 14 days	0.000 *	0.012	(0.001, 0.061)
Sex (male)	0.275	0.709	(0.380, 1.313)
QAIPPE 1	Reference	Reference	Reference
QAIPPE 2	0.102	0.371	(0.110, 1.201)
QAIPPE 3	0.553	0.727	(0.247, 2055)
QAIPPE 4	0.531	0.705	(0.230, 2.075)
QAIPPE 5	0.318	0.604	(0.216, 1.584)
Age Category			
<1 year	Reference	Reference	Reference
13 months to 2 years	0.551	0.561	(0.081, 3.789)
2 to 5 years	0.153	3.451	(0.600, 19.161)
5 to 8 years	0.351	2.243	(0.390, 12.441)
8 to 13 years	0.781	1.268	(0.225, 6.821)
>13 years	0.197	3.139	(0.529, 18.150)

* = significant at 0.05.

**Table 4:** Generalized estimating equations of quality of life following an injury.

	Corrected Quasi Likelihood under Independence Model Criterion (QICC)		
Predictor of interest	PedsQL Total	PedsQL Physical	PedsQL Psychosocial
PaedsCTAS	87274.97 ^†^	169723.43 *	78925.41 ^†^
Hospitalization Status	86231.75 *	164000.67 *	79035.10 *
LOS	82661.30 *	159269.90 *	76715.95 *

^†^ Not significant; * = significant at 0.05; *n* = 185.
